# NMR in a crystallography-based high-throughput protein structure-determination environment

**DOI:** 10.1107/S1744309110030320

**Published:** 2010-09-30

**Authors:** Kurt Wüthrich

**Affiliations:** a Department of Molecular Biology and Skaggs Institute of Chemical Biology, The Scripps Research Institute, La Jolla, CA 92037, USA; b Joint Center for Structural Genomics, http://www.jcsg.org, USA

**Keywords:** NMR in structural genomics, NMR structures of proteins, NMR and crystal structure comparison

## Abstract

An introduction is provided to three papers which compare corresponding protein crystal and NMR solution structures determined by the Joint Center for Structural Genomics (JCSG). Special mention is made of the JCSG strategy for combined use of the two techniques, and of potential applications of the concept of ‘reference crystal structures’, which is introduced in the following three papers.

## Abstract 2

As an introduction to three papers on comparisons of corresponding crystal and NMR solution structures determined by the Joint Center for Structural Genomics (JCSG), an outline is provided of the JCSG strategy for combined use of the two techniques. A special commentary addresses the potentialities of the concept of ‘reference crystal structures’, which is introduced in the following three papers.

The NMR Core of the Joint Center for Structural Genomics (JCSG) has devoted a large part of its work to efficient high-quality NMR structure determination of small soluble proteins based on the recording of extensive networks of nuclear Overhauser effect (NOE) upper limit distance constraints. This effort is an alternative to other projects pursued under the auspices of the Protein Structure Initiative (PSI; see, for example, Cornilescu *et al.*, 2007 ▶; Liu *et al.*, 2005 ▶) and is complementary to PSI projects that are focused on obtaining NMR structures of proteins from a minimal amount of experimental data (see, for example, Raman *et al.*, 2010 ▶; Shen *et al.*, 2009 ▶). The protocol followed in our approach makes use of the software *UNIO* (Herrmann *et al.*, 2002*a* ▶,*b* ▶; Fiorito *et al.*, 2008 ▶; Volk *et al.*, 2008 ▶) for extensive automation of NMR structure determination. It further emphasizes that all data collection for a given protein is performed under identical solution conditions and that the structural information is obtained from a minimal number of NMR experiments, *i.e.* APSY-NMR (Hiller *et al.*, 2005 ▶, 2008 ▶) for polypeptide backbone assignments and three-dimensional heteronuclear-resolved [^1^H,^1^H]-NOESY for side-chain assignments and collection of conformational constraints. In the context of validating the results of this new approach against high-quality crystal data (Brown & Ramaswamy, 2007 ▶), a series of NMR structure determinations were performed for proteins for which a high-resolution crystal structure had previously been determined by the JCSG. In the following three papers, we present com­parisons of the crystal and NMR structures for a selection of five of these proteins. Thereby, once it had been established that the two methods yielded near-identical global molecular architectures, we further investigated possible complementarities of the results from the two techniques.

Over the years, much effort by many different groups has been devoted to deriving the behavior of protein molecules in solution or other physiological environments from crystallographic data. Examples include the representation of crystal structures by a bundle of conformers (DePristo *et al.*, 2004 ▶), computational prediction based on comparison of NMR and X-ray data (Yang *et al.*, 2007 ▶), combination of multiple crystallographic data sets collected at ambient temperature with and without bound ligands (Fraser *et al.*, 2009 ▶) and supplementing crystal structures with NMR measurements of the frequencies of dynamic processes (Boehr *et al.*, 2010 ▶).

Here, the individual crystal structures were solved by the JCSG at 100 K to about 1.8 Å resolution, whereas the corresponding NMR structures were determined in solution at ambient temperature. Despite the large differences in experimental conditions, the NMR structures could be superimposed with the crystal structures with r.m.s.d. values of <1.0 Å for the backbone heavy atoms. This provided a starting platform for detailed studies of local structure variations and for investigating whether such differences arise from either of the two methods used or from the different chemical environments in solution and in the crystal.

We further explored the use of ‘reference structures’ to support structure comparisons. These were computed using the NMR software with input of upper-limit distance constraints derived from the molecular models that represent the results of the structure determinations by NMR and by X-ray diffraction, respectively. Details of the determination of reference crystal structures and reference NMR structures are described in Jaudzems *et al.* (2010 ▶), and applications have been made to all of the proteins in the three papers. From the combined observations with the different proteins, there is an indication that the concept of reference crystal structures computed with NMR structure-determination software could be an efficient and inexpensive alternative for deriving information on the solution behavior of proteins for which a crystal structure is available.

At the present state of the project, we conclude that the reference-structure approach can build bridges between crystal and solution conformational states primarily because the input derived from the experimental structure for calculating the reference crystal structure consists exclusively of intramolecular conformational constraints. Furthermore, small-molecule ligands from the mother liquor, which in the absence of function-related substrate analogs or effector molecules are often observed in active sites and other protein surface locations in crystals, are not part of the input for the calculation of the reference crystal structure. While these additives to the mother liquor may play critical roles in obtaining high-quality crystals, they typically achieve this desirable effect by locking conformational ensembles into unique structural features. In the reference crystal structures, all local features that are locked either by protein–protein or protein–small ligand contacts in the crystal are by design ‘unlocked’ and the use of the NMR software for structure determination and refinement in explicit water was then found to generate structures that displayed very similar features to those calculated from input data measured by NMR in solution. Attractive traits of the reference crystal structure approach for interpreting experimental crystal structures in terms of their solution characteristics include (i) the computational techniques used are well established, efficient and inexpensive, (ii) reference crystal structures can be generated for larger proteins than are readily accessible to NMR structure determination in solution and (iii) selected intermolecular constraints from specific binding of substrate or effector molecules can readily be re-introduced in future studies.

Whereas the paper by Jaudzems *et al.* (2010 ▶) introduces the tools used for systematic structure comparisons, the paper by Mohanty *et al.* (2010 ▶) applies these tools to proteins that have multiple molecules in the crystal asymmetric unit. The results of this study seem to indicate that information on solution behavior might also be obtained from comparison of multiple molecular structures in the asymmetric crystal unit. Finally, the paper by Serrano *et al.* (2010 ▶) applies the comparison tools to proteins with functional annotation and investigates the complementarity of low-temperature crystal data and NMR solution data for investigation of protein active sites. The work with these functionally annotated proteins leads to the intriguing indication that combined analysis of crystal and solution data might be a promising avenue towards identification of putative active-site regions in domains of unknown function (DUFs).

In the JCSG strategy for the combined use of crystal and NMR structure determination, NMR in solution was assigned the primary task of ‘filling gaps’ whenever the crystallography-oriented high-throughput pipeline failed to produce structures of proteins representing new protein families. The three papers in this section would now appear to indicate that combined use of high-resolution crystal and NMR structure determination may yet be an additional strategy for making good use of the potentialities of the two techniques, adding new value to crystallographic *B* values and r.m.s.d.s among bundles of NMR conformers. There is much promise in this approach with regard to the imminent novel challenges of the newly established NIH NIGMS program ‘PSI:Biology’ (http://www.nigms.nih.gov/Initiatives/PSI/psi_biology/). It seems clear that important new information and insights can result if high-quality structures are generated by both techniques and, considering the high efficiency of structure determination resulting from PSI-1 and PSI-2, this would seem to be a tractable problem.
